# Association of periodic limb movements during sleep and Parkinson disease: A retrospective clinical study

**DOI:** 10.1097/MD.0000000000018444

**Published:** 2019-12-20

**Authors:** Shang-Rung Hwang, Sheng-Wei Hwang, Jin-Cherng Chen, Juen-Haur Hwang

**Affiliations:** a Taichung Girl's Senior High School; b Da-Yeh Junior High School, Taichung; c Departments of Neurosurgery, Dalin Tzu Chi Hospital, Buddhist Tzu Chi Medical Foundation, Chiayi; d School of Medicine, Tzu Chi University, Hualien; e Department of Otolaryngology-Head and Neck Surgery, Dalin Tzu Chi Hospital, Buddhist Tzu Chi Medical Foundation, Chiayi; f Department of Medical Research, China Medical University Hospital, China Medical University, Taichung, Taiwan.

**Keywords:** periodic limb movements during sleep, Parkinson disease, polysomnography

## Abstract

Both of periodic limb movements during sleep (PLMS) and Parkinson disease (PD) were related with dopaminergic system dysfunction. We aimed to investigate the detailed association of PLMS severity and PD.

Clinical and overnight polysomnographic data of 2230 adults older than 40 from a community hospital between November 2011 and June 2017 in Taiwan were collected retrospectively. The association of PLMS severity and PD was analyzed by Fisher exact test, univariate, and multivariate logistic regression.

The mean age was 55.6 years old (standard deviation = 9.8, range = 40–91) for all subjects. There were 2205 subjects without PD and 25 subjects with PD in this study. The distribution of PLMS severity was not significantly different between subjects without PD and with PD (Fischer exact test, *P* = .215). Also, PLMS was not significantly associated with PD using univariate and multivariate logistic regression.

The PLMS severity was not associated with PD.

## 1 Introduction

Parkinson disease (PD) is the 2nd most common neurodegenerative disorder affecting older adults, characterized by the resting tremor, rigidity, bradykinesia, postural instability, and some nonmotor features.^[1,2]^ Excessive daytime sleepiness and sleep abnormalities are also prevalent in patients with PD.^[3]^ Factors contributing to sleep disturbances in patients with PD include the depression or anxiety disorders, dementia, sleep motor disorders, and/or side effects of medications for PD.^[4]^

The PD often comorbided with some noctural movement disorders,^[5]^ and possibly shared common pathophysiologic mechanisms. Some studies had shown that rapid eye movement (REM) sleep behavior disorder might appear more than 10 years before the motor signs of PD. The high prevalence of restless leg syndrome (RLS) in PD and the good response to dopamine receptor agonists suggested a relationship between these 2 diseases.^[6]^

As for periodic limb movements during sleep (PLMS), it is presented with repetitive stereotyped movements, typically in the lower limbs, during sleep. The pathophysiology of PLMS was reported to be related with dysfunction of calcium channels and/or dopaminergic system.^[7]^ PLMS could disrupt sleep and is related with excessive daytime sleepiness.^[8]^ PLMS has been proposed to be associated with increased risk of heart diseases and/or cardiovascular events.^[9]^ Patients with PLMS also have a higher risk for coronary artery disease and cardiovascular diseases, but not acute myocardial infarction (AMI).^[10]^ But, the relationship between PLMS and PD is not very valid, although PLMS is commonly seen in patients with PD.^[5]^

Many animals and human studies have hypothesized that hypoxia and/or its related inflammation were some of the underlying mechanisms for most neurodegeneration diseases, including PD.^[11–17]^ Meanwhile, these PLMS-related complications and/or sequelae might also contribute to neural hypoxia and neuroinflammation and subsequently increase the risk of PD development. Thus, it is reasonable to hypothesize that PLMS might be associated with PD. To our knowledge, however, the detailed association of PLMS severity and PD is never reported till now. Therefore, we aimed to examine this issue by a cross-sectional study.

## 2 Methods

From November 2011 to June 2017, clinical and overnight polysomnography (PSG) data of 2230 adult patients older than 40 at Dalin Tzu Chi Hospital were retrospectively collected. The study was approved by The Research Ethics Committee of Dalin Tzu Chi Hospital, Buddhist Tzu Chi Medical Foundation (no: B10604018). Informed written consent was waived because the study was a retrospective data analysis.

Clinical data including age, sex, body mass index (BMI), insomnia, Epworth sleepiness scale (ESS), hypnotic use, apnea-hyponea index (AHI), common systemic diseases, menopause, smoking, and drinking were acquired before overnight PSG examination. Insomnia, hypnotic use, common systemic diseases, menopause, smoking, and drinking were graded as “no” and “yes.” As for PLMS, it was treated by 2 methods. First, PLMS was originally a continuous variable (/h). Second, PLMS was graded into 4 grades as “no,” “mild (≥5/h but <25/h),” “moderate (≥25/h but <50/h),” and “severe (≥50/h).” And, PD was graded as “no” and “yes,” and regarded as non-PD group and PD group, respectively.

### 2.1 Statistical analysis

Student *t* test or Fisher exact test was used to test the association of various clinical factors including PLMS and PD. Multivariate logistic regression was used to test the association of PLMS and PD with adjustment of other promising clinical factors (*P* > .2 from univariate logistic regression). All analyses were performed using STATA 10.0 software (Stata Corp, College Station, TX). *P* < .05 was considered to be significant.

## 3 Results

The mean age was 55.6 years old (standard deviation = 9.8, range = 40–91) for all subjects. Among that, there were 2205 subjects without PD and 25 subjects with PD. Table 1 shows the comparisons between non-PD subjects and PD subjects. Mean age was older in PD subjects (67.1 years old) than in non-PD subjects (55.4 years old). The prevalences of hypnotic use (64.0% vs 38.6%), AMI (16.0% vs 2.8%), stroke (20.0% vs 2.4%), and dementia (16.7% vs 0.2%) were higher in PD subjects than in non-PD subjects. But, the distribution of PLMS severity was not significantly different between non-PD and PD subjects.

**Table 1:**
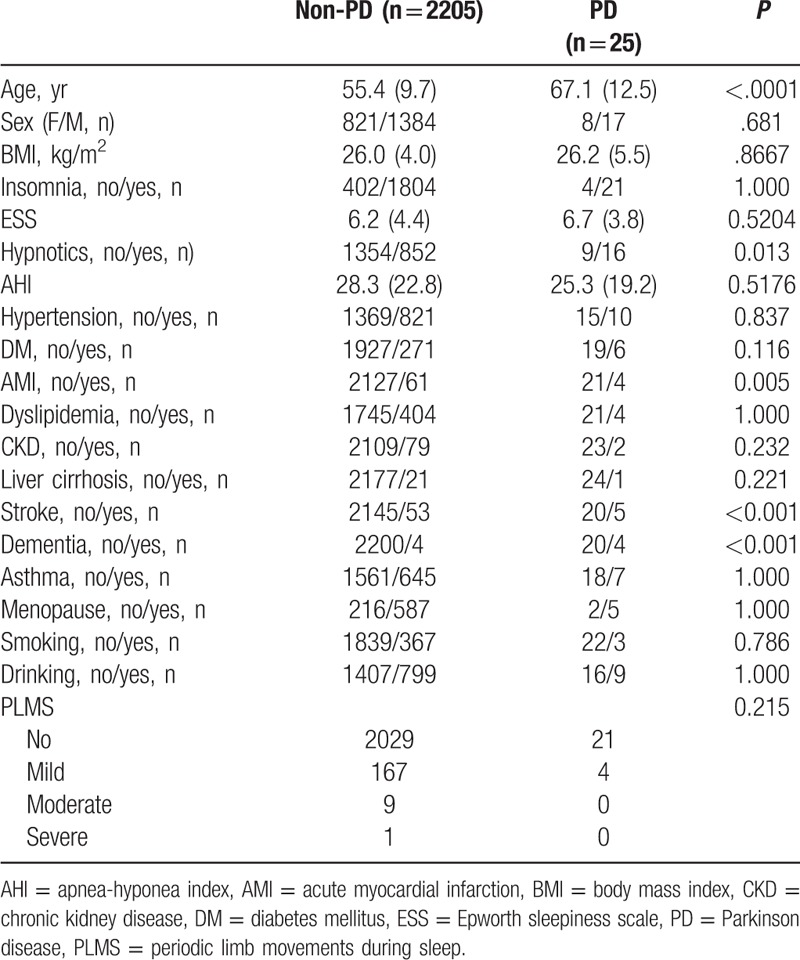
Comparisons between non-PD and PD subjects.

Table 2 shows the univariate logistic regression analysis for the association of various clinical factors and PD. The results showed that age, hypnotics use, AMI, stroke, and dementia, but not PLMS, had significantly positive association with PD individually.

**Table 2:**
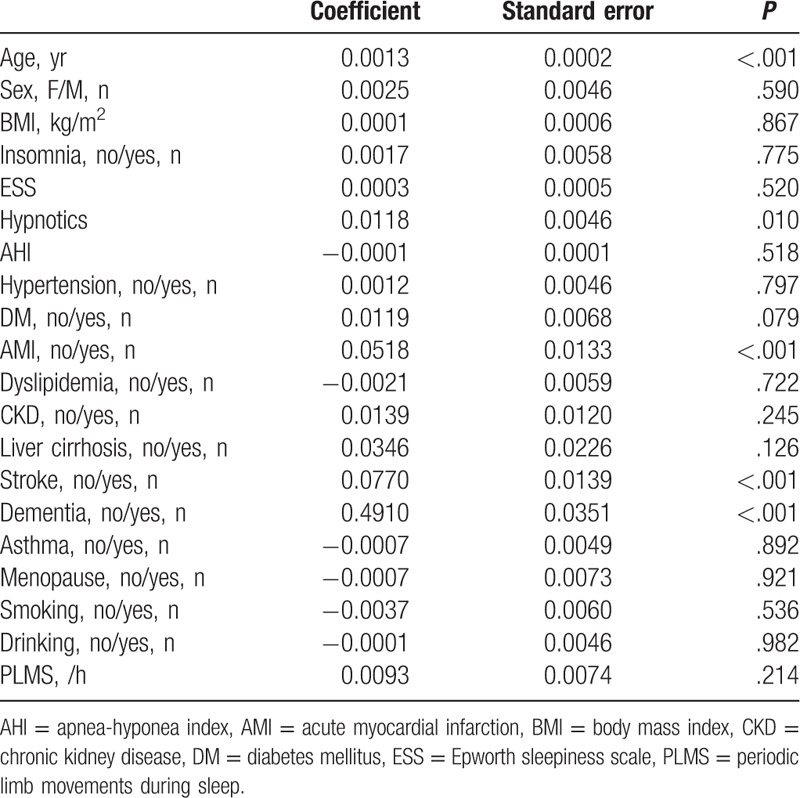
Univariate logistic regression for Parkinson disease by each variables.

Table 3 shows the multivariate logistic regression analysis for the association of PLMS and PD with adjustment of prominent clinical factors. The results showed that PLMS was not significantly associated with PD after adjusting for age, hypnotics use, diabetes mellitus (DM), AMI, liver cirrhosis, stroke, and dementia.

**Table 3:**
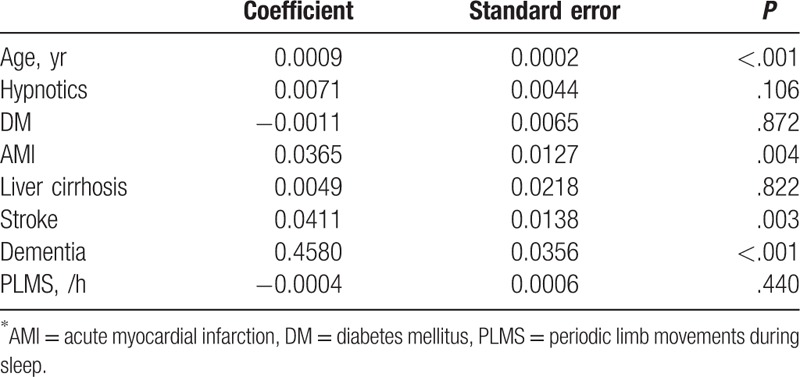
Multivariate logistic regression for tinnitus by prominent variables (*P* < .2).

## 4 Discussion

This cross-sectional study based on clinical and PSG data showed that severity of PLMS, one kind of sleep movement disorders, was not significantly associated with PD before and after adjusting other variables. This clinical finding did not support the expectation based on the dopaminergic system dysfunction for both of PLMS and PD.^[7]^ Also, although PLMS-related complications and/or sequelae were supposed to contribute to PD formation indirectly, current results did not show significantly positive correlation between PLMS and PD at all.

Nocturnal disturbances of PD could be categorized in 4 groups: PD-related motor symptoms; treatment-related nocturnal disturbances; psychiatric symptoms; other sleep disorders, including insomnia, REM behavioral disorder RBD, RLS, PLMS, and excessive daytime sleepiness (EDS).^[18]^ Sleep fragmentation, sleep apnea, RLS, PLMS, and REM sleep behavior disorder may reflect the anatomic areas affected by the neurodegenerative process.^[19]^

As for their treatment, RLS and PLMS may be controlled by increasing dopaminergic and/or α2δ calcium channel stimulation, whereas insomnia and EDS may be improved by reducing dopaminergic stimulation.^[7,18]^

Although RLS and PLMS are different by definition,^[20]^ their treatments are very similar.^[21]^ RLS and/or PLMS might also increase cardiovascular risk by increasing sympathetic tones and sleep fragmentation.^[22]^ PLMS are also commonly found in patients with OSA.^[23]^ And, OSA and PLMS might contribute to arterial stiffness additively.^[24]^ But, there was no evidence to prove that patients with RLS had an increased risk of suffering from PD.^[6]^ Currently, overnight PSG is the gold standard for quantifying the severity of PLMS. However, we found that PLMS severity had no significant association with PD. Otherwise, PLMS might increase the risk of PD indirectly via its comorbid diseases, including OSA as our previous study had shown.^[25]^

The PD affects 2% to 3% of the population older than 65 years of age and its prevalence increases with age.^[26]^ Although the mean age was higher in the non-PD group roughly in Table 1, we have adjusted the effect of age as well as other confounding factors during testing the association of PLMS and PD. However, some limitations were recognized in this study. For example, the data were collected from clinical samples instead of the general population, which could have led to a selection bias. Some factors, including medical psychiatric disorders, were not recorded and accessed in the analysis.

## 5 Conclusion

This clinical study found that PLMS severity was not associated with PD even though there was similar pathophysiology for these 2 diseases. However, this finding did not against the high prevalence of PLMS in the PD population, as previous studies showed.

## Author contributions

**Conceptualization:** Shang-Rung Hwang, Sheng-Wei Hwang, Juen-Haur Hwang.

**Data curation:** Shang-Rung Hwang, Sheng-Wei Hwang, Juen-Haur Hwang.

**Formal analysis:** Juen-Haur Hwang.

**Methodology:** Shang-Rung Hwang, Sheng-Wei Hwang.

**Supervision:** Jin-Cherng Chen.

**Writing – original draft:** Shang-Rung Hwang, Sheng-Wei Hwang.

**Writing – review & editing:** Juen-Haur Hwang.
